# Farnesol Ameliorates Demyelinating Phenotype in a Cellular and Animal Model of Charcot-Marie-Tooth Disease Type 1A

**DOI:** 10.3390/cimb43030138

**Published:** 2021-11-13

**Authors:** Na-Young Park, Geon Kwak, Hyun-Myung Doo, Hye-Jin Kim, So-Young Jang, Yun-Il Lee, Byung-Ok Choi, Young-Bin Hong

**Affiliations:** 1 Department of Translational Biomedical Sciences, Graduate School of Dong-A University, Busan 49201, Korea; skdud1231@naver.com; 2 Department of Health Sciences and Technology, SAIHST, Sungkyunkwan University, Seoul 06351, Korea; kgun903@naver.com (G.K.); gusaud0119@naver.com (H.-M.D.); rgw1313@naver.com (H.-J.K.); 3 Departments of Biochemistry, College of Medicine, Dong-A University, Busan 49201, Korea; soung3e@dau.ac.kr; 4 Well Aging Research Center, Division of Biotechnology, Daegu Gyeongbuk Institute of Science and Technology (DGIST), Daegu 42988, Korea; ylee56@dgist.ac.kr; 5 Samsung Medical Center, Department of Neurology, Sungkyunkwan University School of Medicine, Seoul 06351, Korea

**Keywords:** Charcot-Marie-Tooth disease (CMT), farnesol, myelination

## Abstract

Charcot-Marie-Tooth disease (CMT) is a genetically heterogeneous disease affecting the peripheral nervous system that is caused by either the demyelination of Schwann cells or degeneration of the peripheral axon. Currently, there are no treatment options to improve the degeneration of peripheral nerves in CMT patients. In this research, we assessed the potency of farnesol for improving the demyelinating phenotype using an animal model of CMT type 1A. In vitro treatment with farnesol facilitated myelin gene expression and ameliorated the myelination defect caused by *PMP22* overexpression, the major causative gene in CMT. In vivo administration of farnesol enhanced the peripheral neuropathic phenotype, as shown by rotarod performance in a mouse model of CMT1A. Electrophysiologically, farnesol-administered CMT1A mice exhibited increased motor nerve conduction velocity and compound muscle action potential compared with control mice. The number and diameter of myelinated axons were also increased by farnesol treatment. The expression level of myelin protein zero (MPZ) was increased, while that of the demyelination marker, neural cell adhesion molecule (NCAM), was reduced by farnesol administration. These data imply that farnesol is efficacious in ameliorating the demyelinating phenotype of CMT, and further elucidation of the underlying mechanisms of farnesol’s effect on myelination might provide a potent therapeutic strategy for the demyelinating type of CMT.

## 1. Introduction

Charcot-Marie-Tooth disease (CMT) is a heterogeneous group of inherited diseases affecting the peripheral nervous system [1,2]. Due to the degeneration of the peripheral nerve, CMT patients exhibit progressive and symmetrical distal weakness, as well as gait disturbance [3,4]. Pathologically, CMT is divided into two types: a demyelinating type and an axonal type [3,5]. The pathomechanisms of the demyelinating type are mainly attributed to the aberrant function of myelin genes in Schwann cells, and those of the axonal type are associated with the detrimental effects of mutant proteins on the peripheral axons. Genetically, more than 100 genes have been reported to affect the integrity of Schwann cells or to induce the degeneration of peripheral axons [6,7].

To date, various pathomechanisms of CMT have been proposed for each causative gene. In the axonal type of CMT, mutations in the causative genes cause axonal degeneration by affecting various cellular activities [8]. For example, defects in axonal and endosomal trafficking are caused by the mutations in *RAB7*, *DCTN1*, *KIF1A*, heat shock protein 22 kD (HSP22), and heat shock protein 27 kD (HSP27) [9,10,11,12,13]. Mutations in mitofusin 2 (MFN2), ganglioside-induced differentiation associated protein-1 (GDAP1), and optic atrophy-1 (OPA1) affect mitochondrial dynamics [14,15,16]. Additionally, recent discovery of multiple aminoacyl-tRNA synthetase (ARS) genes, such as glycyl-ARS (GARS) and tyrosyl-ARS (YARS), have also issued that impairment of mRNA processing and protein translation cause peripheral neuropathic phenotype [17,18,19]. On the other hand, mutations in myelinating proteins or their transcription factors affect Schwann cell myelination. Overexpression or mutation of peripheral myelin protein 22 (*PMP22*), as well as myelin protein zero (MPZ), can induce endoplasmic reticulum (ER)-stress-mediated pathogenesis in myelinating Schwann cells [20,21]. However, the precise pathological mechanisms for each causative gene are largely unknown [22].

So far, various therapeutic approaches have been proposed to ameliorate the neuropathic symptoms of CMT [23,24,25,26,27]; however, no satisfactory clinical outcomes have yet been achieved [28]. Therefore, the development of novel therapeutic approaches based on the specific pathomechanisms of each causative gene are required.

In this study, we investigated the feasibility of farnesol as a molecular therapeutic for the demyelinating type of CMT. Farnesol, an acyclic sesquiterpene isoprenol, has been reported to modulate oxidative stress and inflammation through reductions in iNOS, TNF-α, and IL-1ß [29,30]. In addition, farnesol showed protective effects against neurodegenerative disease models and mitigated reactive gliosis in acrylamide-induced neurodegeneration [31,32]. It also significantly delayed the onset and decreased the disease severity of an experimental autoimmune encephalomyelitis (EAE) model [33]. Thus, we evaluated the therapeutic efficacy of farnesol for treating the demyelinating type of CMT using a CMT1A mouse model.

## 2. Materials and Methods

### 2.1. Cell Culture

Rat Schwann cells, RT4 (RT4-D6P2T, CRL-2768, ATCC; Manassas, VA, USA), were cultured in 10% fetal bovine serum, 1% penicillin-streptomycin solution (5000 units/mL penicillin, 5 mg/mL streptomycin), and low-glucose Dulbecco’s modified eagle medium (Biowest, Nuaille, France). RT4 cells (1 × 10^4^) grown for 24 h in 12-well plates, transfected with either control and *PMP22*-containing vectors, pCMV-myc and pCMV-myc-*PMP22* [34], respectively, using Lipofectamine 3000 reagent (Invitrogen, Paisley, UK) in accordance with the manufacturer’s protocol. Three hour after transfection, farnesol (Sigma, St. Louis, MO, USA) were treated onto the cells. To determine the effective concentration of farnesol, we have tested from 10 nM to 10 μM of farnesol, and 0.1 μM of farnesol was used in the data. The effect on the cell viability/proliferation was determined by direct counting under a microscope after 24, 48, and 72 h after farnesol treatment. To reduce the transfection variation, we performed at least three independent experiments.

### 2.2. Quantitation of mRNA

To quantify the mRNA levels of myelination genes, total mRNA was prepared using an RNeasy Mini Kit (Qiagen, Hilden, Germany). Then, cDNA was synthesized with SuperScript^TM^ II reverse transcriptase (Invitrogen), oligo-dT for primer, and the isolated total mRNA as a template according the manufacturer’s instruction. Then, real-time PCR amplification was performed with the cDNA, SYBR Green PCR master mix, ABI 7900 (Applied Biosystems, Warrington, UK), and the following primers: β-actin-forward (F), 5′-GAAAACAGCAGCAGTGACCA-3′; β-actin-reverse (R), 5′-CTCTCAGCTGTGGTGGTGAA-3′; Oct6-F, 5′-GTTCTCGCAGACCACCATCT-3′; Oct6-R, 5′-CCTTTGACACCCACCTCAAT-3′; MPZ-F, ′5-CTGGTCCAGTGAATGGGTCT-3′; and MPZ-R, 5′-CATGTGAAAGTGCCGTTGTC-3′. Determination of gene expression was performed after the average Ct value of each gene was normalized to that of β -actin. Then, the expression level of each gene was calculated using the formula 2^−(meanΔΔCt)^.

### 2.3. Mouse Drug Administration and Phenotype Evaluation

C22 mice (B6;CBACa-Tg(*PMP22*)C22Clh/H) obtained from MRC Harwell (Oxfordshire, UK) were used as a CMT1A model [35]. As the C22 mouse, that harbors seven copies of human *PMP22* genes, exhibits CMT1A symptom in a heterozygous for transgene, all the experiments were performed using heterozygous C22 mice. Optimal route and dose of farnesol administration was determined from previous works [36,37], and the administration was performed from 3 weeks of age until the end of the experiments (13 weeks of age). The C22 mice in the farnesol-treated group (*n* = 10, 5 males and 5 females) were fed an AIN-76A diet containing 0.5% (*w*/*w*) farnesol, and control group (*n* = 10, 5 males and 5 females) were fed an AIN-76A diet. In addition, equal numbers of wild-type (*n* = 10, 5 males and 5 females) were also included as a reference group. All experiments were conducted according to protocols approved by the Institutional Animal Care and Use Committees of Samsung Medical Center (protocol code 20150702003).

Evaluations of peripheral neuropathic phenotypes were performed according to a previous study [38,39]. Briefly, assessments of motor coordination and balance were performed using an apparatus with a horizontal rod rotating at 21 rpm (B.S. Technolab Inc., Seoul, Korea). The maximum time the mice held onto the rotating rod was recorded for up to 180 s. A nerve conduction study was performed to determine motor nerve conduction velocity (MNCV) and compound muscle action potential (CMAP) using a Nicolet VikingQuest device (Natus Medical, San Carlos, CA, USA) after the mice were anesthetized with 1.5% isoflurane supplied using a nose cone.

### 2.4. Electron Microscopy and Determination of Axon Diameter

The sciatic nerves were biopsied at the end of the experiments, and the specimens were fixed in 2% glutaraldehyde in 25 mM cacodylate buffer. Semi-thin sections were stained with toluidine blue, and ultra-thin cut samples were stained for 10 min with 1% uranyl acetate and Reynold’s lead citrate. The specimens were observed with an HT7700 electron microscope (Hitachi, Tokyo, Japan) at 80 kV. The axon diameter was determined using the Zeiss Zen2 program (Carl Zeiss, Oberkochen, Germany) in accordance with previously reported methods [38].

### 2.5. Immunohistochemistry

The sciatic nerves were isolated and fixed in 4% PFA for 24 h, then the tissues were cryoprotected in a 20% sucrose solution. Cross-serial sections (10 μm) were prepared using a cryostat (Leica, Nussloch, Germany); then, the slides were permeabilized with cold methanol and washed with PBS followed by blocking with 5% FBS and 0.3% Triton X-100 in PBS for 1 h. The sections were incubated with a primary antibody against MPZ (Abcam, Cambridge, UK) or NCAM (R & D Systems, Minneapolis, MN, USA) for 16 h at 4 °C, followed by incubation with Alexa 488 or Cy3-conjugated secondary antibody (Sigma) for 2 h. After coverslips adhered to the glass slides with mounting medium (Biomeda, Foster City, CA, USA), images were obtained under a Zeiss imager M2 with an ApoTome II microscope (Carl Zeiss). The intensity of the immunofluorescent staining was measured over an area of 500 × 600 μm^2^ using Zen 2 Blue edition software.

### 2.6. Statistical Analysis

All statistical analyzes and data visualization were performed in Prism 8.1.0 (GraphPad Software, San Diego, CA, USA). The significance of the data was evaluated by one-way ANOVA with Tukey’s post-hoc multiple comparison, and the results were expressed as the mean and standard error of the mean (SEM). *p* values < 0.05 were considered statistically significant.

## 3. Results

### 3.1. Farnesol Increases Myelination In Vitro

**Figure 1:**
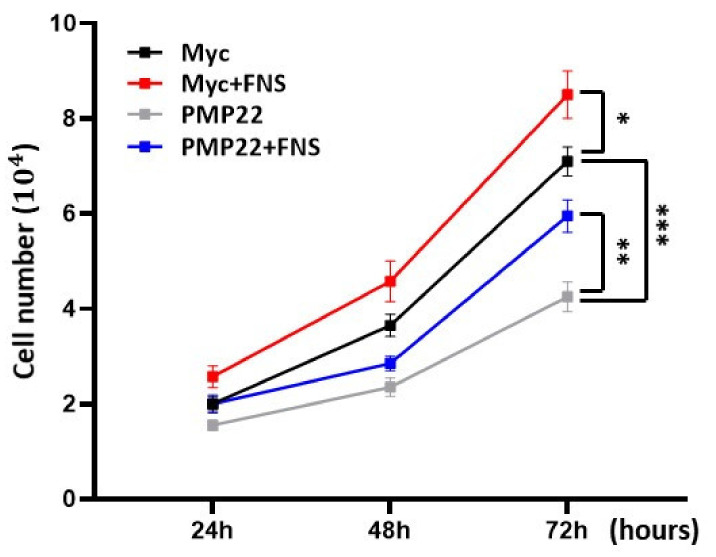
Farnesol increases cell proliferation. RT4 (1 × 10^4^) cells grown in 12-well plates were transfected with pCMV-myc-*PMP22* or pCMV-myc vector with or without 0.1 μM of farnesol. Direct cell counts after farnesol treatment for indicated times. * *p* < 0.05; ** *p* < 0.01; *** *p* < 0.001.

As the overexpression of *PMP22* induces apoptotic cell death, we first assessed whether farnesol increased the cell viability of *PMP22*-overexpressing cells. After transfection of either the pCMV-myc-*PMP22* or pCMV-myc (control) vector, the cells were treated with 0.1 μM of farnesol, and changes to cell morphology were assessed 48 h later. RT4 cells overexpressing the *PMP22* gene showed significantly fewer cell numbers than those transfected with the control vector. Direct cell counts revealed that the proliferation of the *PMP22*-overexpressing cells was significantly reduced compared to the control cells after 72 h (*p* < 0.001). However, treatment with farnesol restored the reduction in cell proliferation in *PMP22*-overexpressing cells, which exhibited a 40% increase compared to untreated *PMP22*-overexpressing cells (*p* < 0.01). Farnesol also significantly increased the proliferation rate in the control-vector-transfected cells (Figure 1). These results infer that farnesol protected against cell death or promoted the proliferation of *PMP22*-overexpressing Schwann cells.

**Figure 2:**
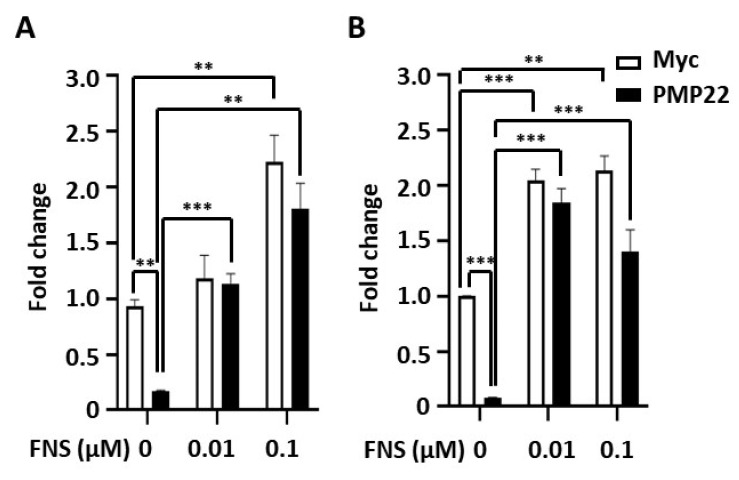
Changes in myelin gene expression by farnesol. After transfection of pCMV-myc-*PMP22* or pCMV-myc vector into RT4 cells, cells were treated with 0.01 μM and 0.1 μM of farnesol for 48 h. (**A**) *Oct6* mRNA levels were determined by RT-PCR. (**B**) Changes in *MPZ* mRNA levels were determined by RT-PCR. ** *p* < 0.01; *** *p* < 0.001.

Next, we addressed whether farnesol promotes myelination of Schwann cells. As demyelination by *PMP22* overexpression is a prominent pathophysiology in CMT1A patients, we investigated whether farnesol restores the myelination process in *PMP22*-overexpressing cells. After transfecting pCMV-myc-*PMP22* into RT4 cells, farnesol was treated for 48 h, and changes in mRNA levels were analyzed. We used Oct6, a transcription factor for myelination, and MPZ, a myelin protein, to determine changes in myelination. The overexpression of *PMP22* reduced the mRNA expression level of *MPZ* and *Oct6* by 92% (*p* < 0.001) and 82% (*p* < 0.01), respectively. In this setting, treatment with farnesol (0.01 μM and 0.1 μM) restored the expression levels of those genes to the levels in the control vector-transfected cells. In addition, farnesol treatment also increased mRNA levels of *Oct6* and *MPZ* in the control cells (Figure 2). These results imply that farnesol promotes the expression of several myelination-related genes in *PMP22*-overexpressing Schwann cells.

### 3.2. Farnesol Ameliorates Demyelinating Phenotype of CMT1A Mouse Model

**Figure 3:**
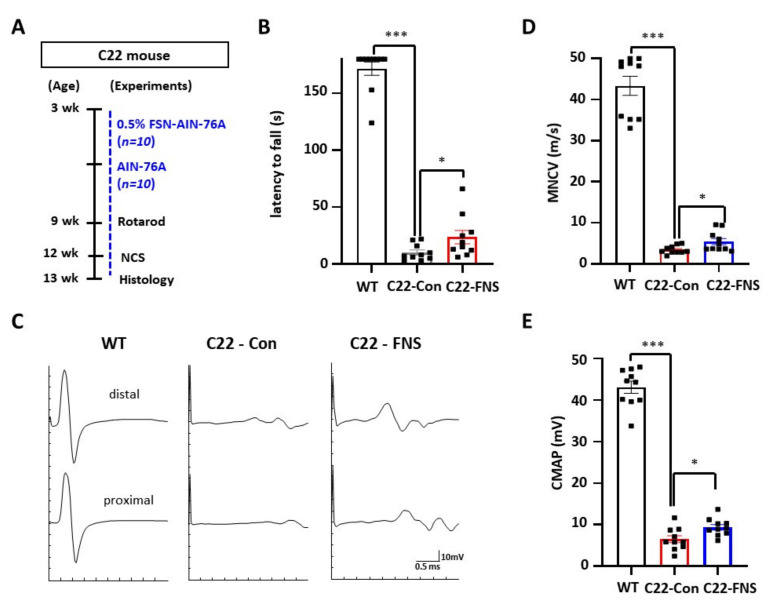
Behavioral and electrophysiological enhancements in farnesol-administered mice. (**A**) Experimental scheme of farnesol administration to C22 mice. C22 mice were fed AIN-76A diet containing 0.5% (*w*/*w*) farnesol (C22-FSN, *n* = 10) or AIN-76A diet (C22-Con, *n* = 10) from 3 weeks of age. Age-matched wild-type mice (*n* = 10) were used as a reference (**B**) Rotarod test was performed to evaluate locomotor function. (**C**) Representative images of electrophysiological evaluation. (**D**) motor nerve conduction velocity (MNCV). (**E**) Compound muscle action potential (CMAP). WT, wild-type mice; C22-Con, control diet fed C22 mice; C22-FNS, farnesol diet fed C22 mice; * *p* < 0.05; and ***, *p* < 0.001.

Because farnesol promoted myelination in Schwann cells, we then assessed whether administration of farnesol ameliorates the demyelinating phenotype of the CMT1A mouse. For in vivo evaluation, we used the C22 mouse model, a well-characterized CMT1A mouse model (Figure 3A).

To assess behavioral improvements in the mice, we measured their rotarod performance. Compared to control C22 mice (10.1 ± 2.2 s), farnesol-fed C22 mice exhibited a significantly increased time (23.6 ± 5.9 s) on the rod (*p* < 0.05) (Figure 3B); thus, farnesol administration improved the locomotor function of C22 mice. Then, an electrophysiological evaluation was undertaken to confirm the amelioration of the demyelinating phenotype (Figure 3C). The MNCV of farnesol-fed C22 mice (5.4 ± 0.5 m/s) was significantly increased compared to that of the control-diet-fed C22 mice (3.4 ± 0.3 m/s) (*p* < 0.05) (Figure 3D). In addition, farnesol-fed C22 mice also exhibited significantly improved CMAP (9.4 ± 0.7 mV) compared to the control C22 mice (6.5 ± 0.8 mV) (*p* < 0.05) (Figure 3E). These data infer that the integrity of the peripheral nerve and Schwann cells were improved by farnesol administration.

**Figure 4:**
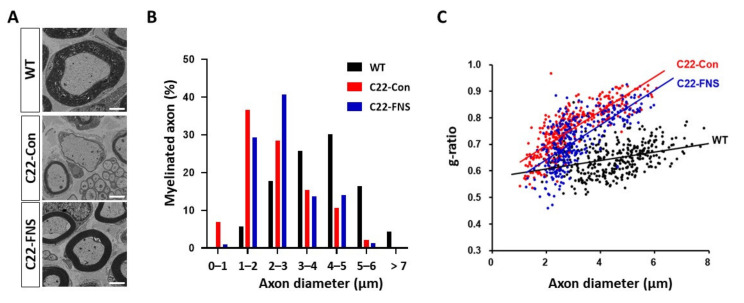
Farnesol improves myelination in sciatic nerves of C22 mice. (**A**) Electron microscopic images of the sciatic nerve. Scale bar, 2 μm. (**B**) Distribution of myelinated axon diameters. Three hundred and twenty to three hundred and fifty axons from 3 mice in each group were counted. (**C**) g-ratio of myelinated axon. WT, wild-type mice; C22-Con, control diet fed C22 mice; C22-FNS, farnesol diet fed C22 mice.

We then directly assessed the myelination of the sciatic nerve. Electron microscopic images revealed that C22 mice exhibited weak myelination with a smaller and thinner myelin sheath in sciatic nerve compared to age-matched wild-type mice. However, these characteristics were improved in farnesol-fed C22 mice (Figure 4A). With further calculations, we determined the distribution of the diameter of the myelinated axons. Compared to wild-type mice (4.488 ± 0.066), C22 mice (2.937 ± 0.063) showed a left-shifted distribution of axon diameters, with the majority being smaller axons. In farnesol-fed C22 mice, the left-shifted distribution was enhanced (3.153 ± 0.057), and their axon diameters were larger than those of the control C22 mice (Figure 4B). In the g-ratio analysis that determines the ratio of myelin thickness and axon diameter, control C22 mice (0.757 ± 0.005) showed a steeper slope compared to the wild-type mice (0.647 ± 0.003), while that of farnesol-fed C22 mice (0.717 ± 0.005) was slightly improved (Figure 4C). In agreement with the behavioral and electrophysiological parameters, these data indicate that farnesol administration improved myelination defects in C22 mice.

**Figure 5:**
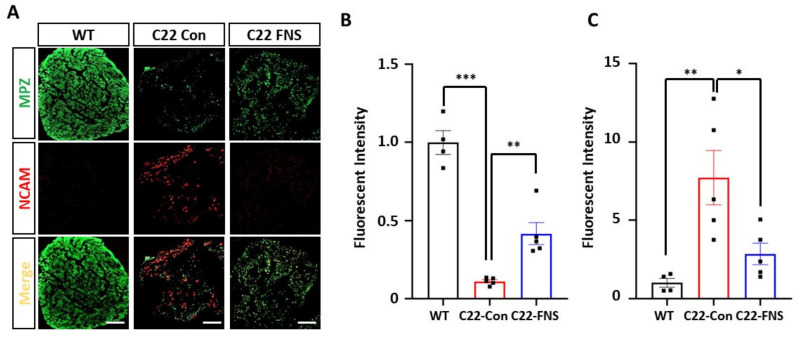
Restoration of sciatic nerve myelination by farnesol. (**A**) Immunofluorescent staining of MPZ and NCAM. Scale bar, 50 μm. (**B**) Fluorescent intensity of MPZ. (**C**) Fluorescent intensity of NCAM. * *p* < 0.05; ** *p* < 0.01; *** *p* < 0.001.

Finally, we assessed whether the histological improvements correlated with functional myelination in the farnesol-fed C22 mice. Using the myelination and demyelination markers, MPZ and NCAM, respectively, we measured the myelination by Schwann cells at the sciatic nerve. Previously, it was reported that the expression level of NCMA correlated with demyelination status in the CMT1A mouse model [40]. Double staining with MPZ and NCAM antibodies revealed that control-diet-fed C22 mice had higher levels of NCAM and lower levels of MPZ compared to wild-type mice (Figure 5A). The level of MPZ expression was significantly higher in wild-type mice than control C22 mice, whereas the level was significantly restored in farnesol-treated mice (Figure 5B). However, the elevated levels of NCAM expression in the control C22 mice were significantly reduced in farnesol-fed C22 mice (Figure 5C). Collectively, these data suggest that farnesol administration dramatically ameliorated the demyelinating phenotype in the CMT1A mouse model.

## 4. Discussion

In these experiments, we evaluated the therapeutic effects of farnesol on the demyelinating type of CMT, which is caused by malfunctions in Schwann cells. Farnesol treatment effectively enhanced the myelinating potential of Schwann cells both in vitro and in vivo. In the CMT1A cell model overexpressing *PMP22*, farnesol significantly increased the mRNA levels of a myelin protein and a related transcription factor. As overexpression of *PMP22* causes demyelination features, such as downregulation of myelin genes, the present data infer that farnesol treatment could improve the demyelinating phenotype. In addition, farnesol treatment restored the reduction in cell proliferation caused by *PMP22* overexpression. As it is well documented that the overexpression of *PMP22* affects the proliferation of Schwann cells and promotes apoptotic cell death [41,42,43,44], our data imply that farnesol treatment improves the abnormal features of Schwann cells.

To validate the findings from the in vitro experiments, we thoroughly investigated the in vivo efficacy of farnesol using a CMT1A mouse model. We found that farnesol treatment increased locomotor function. The amplitude of CMAP, a correlative parameter of muscle integrity [45], also provided evidence for enhanced muscular function by farnesol administration. The elevated level of locomotor function might have been an outcome of the enhanced myelination and axonal integrity. Considering the correlation between the electrophysiological and histological data, the enhanced MNCV infers that myelination is improved by farnesol administration. Indeed, toluidine blue staining and electron microscopic images of the sciatic nerves demonstrated that demyelination was significantly ameliorated by farnesol treatment. Additionally, the functional improvements in Schwann cells apparent on the immunofluorescent images also support the efficacy of farnesol. The level of the myelin protein MPZ was improved, while the expression level of the demyelinating marker, NCAM, was reduced. These parameters also indicated that myelination improvements were positively correlated with axonal integrity. Compared to the wild-type mice, the C22 mice showed a left-shifted pattern in the distribution of myelinated axon diameters, indicating the degeneration of larger axons. Upon farnesol administration, there was an obvious increase in the number of large myelinated fibers. Collectively, these data imply that farnesol treatment ameliorated the demyelinating phenotype by improving the integrity of the peripheral axons and muscles.

In this study, we did not attempt to ascertain the mode of action of farnesol in the promotion of myelination. Previously, farnesol efficacy was reported in treating multiple sclerosis, an inflammatory demyelinating disease of the CNS [33]. In EAE, a well-established model of multiple sclerosis, farnesol treatment delayed the onset of EAE and decreased disease severity. These results might be derived from its immune-modulation effects, a hypothesis supported by the reduced numbers of infiltrating immune cells, such as monocytes/macrophages, dendritic cells, and T cells, in the spinal cord. The immune-modulation or anti-inflammatory effects of farnesol have been suggested by previous reports [36,46]. Farnesol reduced oxidative stress in a cadmium-chloride-induced toxicity model and modulated the release of inflammatory cytokines in an asthma mouse model. In addition, farnesol evidenced neuroprotective effects by modulating the production of free radicals and pro-inflammatory cytokines in Lipopolysaccharides (LPS)-induced or acrylamide-induced mouse models [31,32]. However, these studies did not clearly elucidate the underlying mechanisms by which farnesol causes immune modulation. With regard to CMT pathogenesis, inflammation is not a common feature during the development of the CMT phenotype in patients, although there has been a limited number of reports in patients and animal models [47,48].

Although farnesol possesses some beneficial features of neuroprotection, its direct mechanism of myelination promotion has not been elucidated. According to previous reports, one possible explanation can be hypothesized. Farnesol can modulate Ca^2+^ levels via the inhibition of L-type and N-type Ca^2+^ channels, which regulate cell growth and neurotransmitter release [49,50,51]. A high basal concentration of intracellular calcium was observed in Schwann cells from a CMT1A rat model [52]. A recent study also revealed that *PMP22* is associated with Ca^2+^ influx via its association with stromal interaction molecule 1, the store-operated Ca^2+^ channel subunit in the ER [53]. Thus, the overexpression of wild-type *PMP22* or of mutant *PMP22* can lead to elevated intracellular Ca^2+^ levels, and modulation of intracellular calcium levels might improve the homeostasis of Schwann cells, thereby ameliorating the demyelinating phenotype of CMT.

In this study, we demonstrated that farnesol administration improved the demyelinating phenotype of a CMT1A animal model through the promotion of myelin protein expression. Further studies to elucidate the molecular mechanisms by which farnesol improves Schwann cell homeostasis might provide a novel CMT therapeutic strategy.

## Data Availability

All data generated or analyzed during this study are included in this published article.
